# Higher LDL-C/HDL-C Ratio Is Associated with Elevated HbA1c and Decreased eGFR Levels and Cardiac Remodeling in Elderly with Hypercholesterolemia

**DOI:** 10.3390/jcdd11050140

**Published:** 2024-04-30

**Authors:** Yufeng Li, Gang Li, Jari A. Laukkanen, Linping Wei, Xinrui Chen

**Affiliations:** 1 Division of Cardiology, Department of Geriatrics, The First Affiliated Hospital of Chongqing Medical University, Chongqing 400016, China; yufenglicqmu@126.com (Y.L.); weilinping369@163.com (L.W.); chenxinrui@stu.cqmu.edu.cn (X.C.); 2 Institute of Clinical Medicine, Department of Medicine, University of Eastern Finland, 70211 Kuopio, Finland; jariantero.laukkanen@uef.fi; 3 Institute of Public Health and Clinical Nutrition, University of Eastern Finland, 70211 Kuopio, Finland; 4 Department of Medicine, Wellbeing Services County of Central Finland, 40620 Jyväskylä, Finland

**Keywords:** hypercholesterolemia, glycated hemoglobin, glomerular filtration dysfunction, coronary heart disease, cardiac remodeling

## Abstract

**Background:** This study aims to explore the relationship of the low-density lipoprotein cholesterol (LDL-C)/high-density lipoprotein (HDL-C) ratio with glycated hemoglobin (HbA1c), renal dysfunction, coronary heart disease (CHD) and cardiac structure and function in elderly patients with hypercholesterolemia. **Methods**: A total of 1129 hospitalized Chinese elderly (aged ≥ 65 years) with hypercholesterolemia were collected retrospectively. The patients were divided into low (<2.63), moderate (≥2.63 to <3.33) and high (≥3.33) LDL-C/HDL-C ratio groups according to the tertiles of LDL-C/HDL-C. **Results**: Regression analysis of the LDL-C/HDL-C ratio with metabolic and echocardiographic parameters revealed that a high LDL-C/HDL-C ratio (≥3.33) was associated independently with male gender, elevated HbA1c, decreased estimated glomerular filtration rate (eGFR), prevalent CHD and left ventricular dilatation (all *p* < 0.05). **Conclusions**: A high LDL-C/HDL-C ratio was associated with male gender, increased HbA1c, decreased eGFR, CHD and enlarged left ventricle in elderly with hypercholesterolemia.

## 1. Introduction 

With the aging of the population, the prevalence of dyslipidemia is increasing in China [1]. Increased pro-atherogenic low-density lipoprotein cholesterol (LDL-C) and decreased anti-atherogenic high-density lipoprotein cholesterol (HDL-C) are common abnormalities in dyslipidemia [2,3]. Dyslipidemia plays a crucial role in the occurrence and development of atherosclerotic cardiovascular disease (ASCVD). Elevated LDL-C is a known risk factor for ASCVD, while high total cholesterol (TC) and triglyceride (TG) levels and low HDL-C are also independently related to ASCVD [2]. However, these individual lipid parameters are unable to indicate completely the balance between the pro- and anti-atherogenic lipoprotein profiles. Previous studies have confirmed that the LDL-C/HDL-C ratio, as a comprehensive lipid parameter, was a better predictor for ASCVD compared with the conventional lipid parameters [4,5]. An increased TG/HDL-C ratio was reported to be a predictor of insulin resistance, hyperglycemia and incident type 2 diabetes mellitus (DM) [6,7]. However, studies have shown that a high LDL-C/HDL-C ratio is strongly associated with diabetes [8], chronic kidney disease (CKD) [9] and coronary heart disease (CHD) [4]. In addition, other investigations have demonstrated that a high LDL-C/HDL-C ratio is strongly associated with left ventricular hypertrophy and cardiac death [10,11,12]. Nevertheless, the relevance of the LDL-C/HDL-C ratio to various cardiometabolic parameters and cardiac remodeling is still poorly understood in elderly patients with hypercholesterolemia. Therefore, this study explored the association of the LDL-C/HDL-C ratio with glycated hemoglobin (HbA1c), renal dysfunction, prevalent CHD and cardiac structure and function in the elderly with hypercholesterolemia in greater detail.

## 2. Methods

### 2.1. Study Design and Participants

A cross-sectional study was conducted, in which 1129 hospitalized Chinese elderly (aged ≥ 65 years) with hypercholesterolemia were included by review of medical records from 2014 to 2022 in our inpatient department. All of the patients underwent abdominal imaging, color Doppler echocardiography and biochemical tests including detailed blood lipid profile assessments. 

Hypercholesterolemia was defined as LDL-C ≥ 3.37 mmol/L after fasting for at least 12 h, whether the patients took lipid-modifying drugs or not [13].

The patients with hypercholesterolemia were divided into a low LDL-C/HDL-C ratio group (the ratio < 2.63, *n* = 376), moderate LDL-C/HDL-C ratio group (2.63 ≤ the ratio < 3.33, *n* = 376) and high LDL-C/HDL-C ratio group (the ratio ≥ 3.33, *n* = 377) according to the tertiles of the LDL-C/HDL-C ratio.

We excluded patients with infection; autoimmune disease; glomerulonephritis; acute myocardial infarction (MI) or stroke; flared gout; hypertrophic, restrictive or dilated cardiomyopathy; valvular or congenital heart diseases; atrial fibrillation or flutter; chronic obstructive pulmonary disease; malignant tumors; hereditary hypercholesterolemia; type 1 and other types of DM; secondary hypertension; or fatty liver complicated with viral, autoimmune, alcoholic or hereditary metabolic diseases.

### 2.2. Diagnostic Criteria for Comorbidities

Type 2 DM: fasting plasma glucose (FPG) ≥ 7.0 mmol/L, oral 75 g glucose post-loaded 2 h plasma glucose ≥ 11.1 mmol/L in oral glucose tolerance test and/or HbA1c ≥ 6.5% with normal or increased plasma insulin concentration, or previously diagnosed type 2 DM in which the patients were treated with insulin or oral glucose-lowering medication [14].

Primary hypertension: averaged systolic blood pressure (BP) ≥ 140 mmHg and/or diastolic BP ≥ 90 mmHg after 10 min rest and two measurements of BP, or hypertension was confirmed previously and the patients were taking antihypertensive medication [3].

Non-alcoholic fatty liver was confirmed by ultrasound, computed tomography (CT) or magnetic resonance imaging (MRI) [15].

CHD was defined as a previous history of angina pectoris, MI, coronary artery stenting, or one coronary atherosclerotic stenosis ≥ 50% confirmed by angiography [4].

Stroke was defined as a previous history of hemiparesis, and the relevant brain imaging changes were confirmed by CT or MRI [16].

### 2.3. Measurement of Lipids, Glycated Hemoglobin and High-Sensitivity C-Reactive Protein

Fasting plasma TC and TG levels were assayed enzymatically; HDL-C and LDL-C were measured by homogeneous enzyme colorimetry [17]. HbA1c was detected by high-performance liquid chromatography [18]. High-sensitivity C-reactive protein (hsCRP) was determined by immunoturbidimetry [18].

### 2.4. Calculation of Estimated Glomerular Filtration Rate and Body Mass Index

For men, serum creatinine (Scr) > 80 µmol/L, estimated glomerular filtration rate (eGFR) = 141 × (Scr µmol/L/88.4/0.9)^−1.209^ × 0.993^Age (years)^; Scr ≤ 80 µmol/L, eGFR = 141 × (Scr µmol/L/88.4/0.9)^−0.411^ × 0.993^Age (years)^. For women, Scr > 62 µmol/L, eGFR = 144 × (Scr µmol/L/88.4/0.7)^−1.209^ × 0.993^Age (years)^; Scr ≤ 62 µmol/L, eGFR = 144 × (Scr µmol/L/88.4/0.7)^−0.329^ × 0.993^Age (years)^ [19]. Body mass index (BMI) = body weight (kg)/[height (m)]^2^ [20].

### 2.5. Detection of Cardiac Structure and Function

Transthoracic two-dimensional M-mode echocardiography using a GE Vivid 7 full digital color Doppler ultrasound instrument was used to determine the following parameters: right atrial diameter (RAD), right ventricular diameter (RVD), left atrial diameter (LAD), interventricular septal thickness (IVST), left ventricular posterior wall thickness (LVPWT), end-systolic diameter (LVESD), end-diastolic diameter (LVEDD), ejection fraction (LVEF) and early (E)/late (A) diastolic peak mitral inflow velocity [21].

### 2.6. Statistical Analysis

The statistical package for social science (SPSS) 26.0 software (IBM Company, Chicago, IL, USA) was used for the statistical analysis. The cutoff values of the LDL-C/HDL-C ratio tertiles used in the current study were calculated by the SPSS software, according to the LDL-C/HDL-C values of the elderly subjects in the present study. Continuous data were expressed as mean ± standard deviation ($\bar{x}$ ± *s*); one-way analysis of variance (ANOVA) was used to analyze normally distributed data among multiple groups, and then the LSD test was applied for a comparison between the two groups. If the data were distributed non-normally, the Kruskal–Wallis H test was used to compare the data among multiple groups, and then the Mann–Whitney U test was used for a comparison between two groups. Count data were presented as percentages, and the chi-square test was used. Pearson’s or Spearman’s correlation analysis was used for univariate analysis. Dichotomized logistic regression analysis was performed for multifactorial analysis. Because TC, TG, LDL-C and HDL-C were collinear with the LDL-C/HDL-C ratio, these parameters were not included in the regression analysis. A 2-tailed value of *p* < 0.05 was considered significant statistically.

## 3. Results

### 3.1. Clinical Characteristics According to the Tertiles of the LDL-C/HDL-C Ratio

Compared with the low LDL-C/HDL-C ratio group, the BMI, diabetic prevalence ratio and duration, fatty liver prevalence ratio, HbA1c, LDL-C, TC and TG were higher (all *p* < 0.05) and the HDL-C was lower in the moderate LDL-C/HDL-C ratio group (*p* < 0.05); The prevalence ratio of male gender, smoking, drinking, type 2 DM, hypertension, fatty liver and CHD, BMI, diabetic duration, diastolic BP, HbA1c, LDL-C, TC, TG and hsCRP were higher (all *p* < 0.05) and eGFR and HDL-C were lower in the high LDL-C/HDL-C ratio group (both *p* < 0.05). In contrast to the moderate LDL-C/HDL-C ratio group, the prevalence ratio of male gender, smoking and CHD, HbA1c, LDL-C, TC and TG were higher (all *p* < 0.05), while the eGFR and HDL-C were lower in the high LDL-C/HDL-C ratio group (all *p* < 0.05, Table 1).

Univariate correlation analysis revealed that LDL-C/HDL-C ratio was positively associated with the prevalence ratio of male gender, smoking, alcohol use, DM, hypertension, fatty liver, CHD, BMI, DM history, diastolic BP, HbA1c, LDL-C, TC, TG and hsCRP (all *p* < 0.05), while it was associated negatively with eGFR and HDL-C (both *p* < 0.05, Table 1).

### 3.2. Cardiac Remodeling Corresponding to the Tertiles of the LDL-C/HDL-C Ratio

Compared with the low LDL-C/HDL-C ratio group, the RVD and LAD were larger in the moderate LDL-C/HDL-C ratio group (both *p* < 0.05), while RVD, LAD, LVESD, LVEDD, IVST and LVPWT were larger (all *p* < 0.05) and LVEF was lower in the high LDL-C/HDL-C ratio group (*p* < 0.05). Compared to the moderate LDL-C/HDL-C ratio group, LAD, LVESD, LVEDD, IVST and LVPWT were larger (all *p* < 0.05) and LVEF was lower in the high LDL-C/HDL-C ratio group (*p* < 0.05, Table 2).

Univariate correlation analysis suggested that the LDL-C/HDL-C ratio was positively correlated with RVD, LAD, LVESD, LVEDD, IVST and LVPWT (all *p* < 0.05) and negatively correlated with LVEF (*p* < 0.05, Table 2).

### 3.3. Logistic Regression Analysis of High LDL-C/HDL-C Ratio with Clinical Characteristics and Echocardiographic Parameters

**Figure 1:**
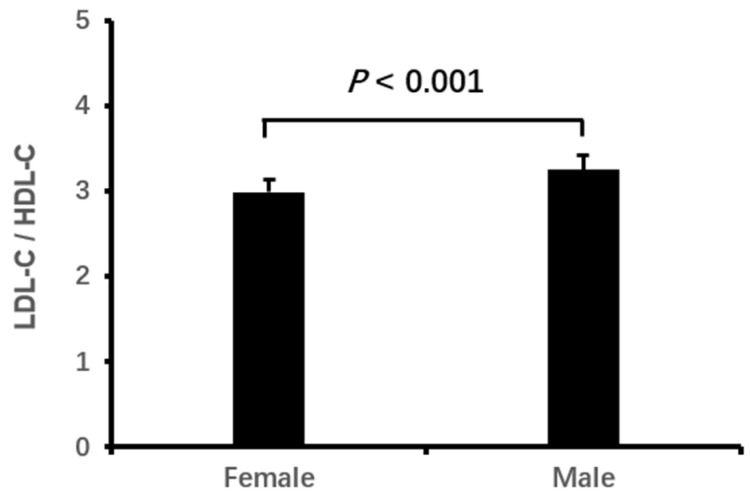
The LDL-C/HDL-C ratio in men and women. LDL-C, low-density lipoprotein cholesterol; HDL-C, high-density lipoprotein cholesterol.

**Figure 2:**
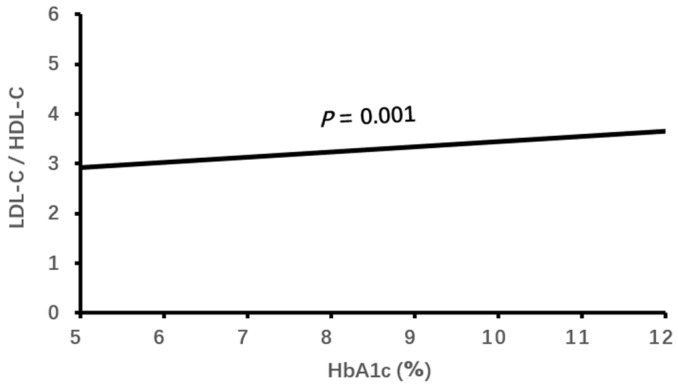
The association of LDL-C/HDL-C ratio with HbA1c. LDL-C, low-density lipoprotein cholesterol; HDL-C, high-density lipoprotein cholesterol; HbA1c, glycated hemoglobin.

**Figure 3:**
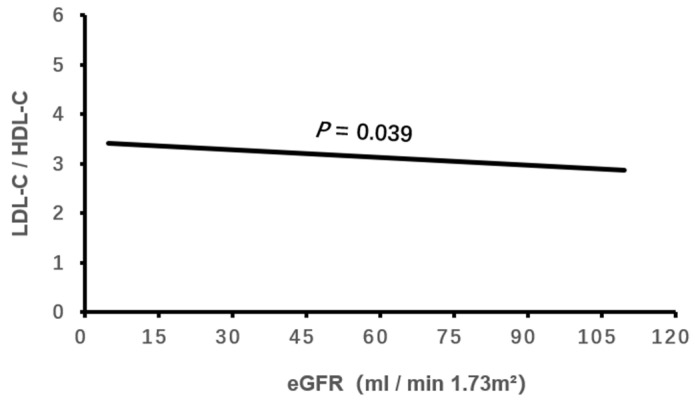
The association of LDL-C/HDL-C ratio with eGFR. LDL-C, low-density lipoprotein cholesterol; HDL-C, high-density lipoprotein cholesterol; eGFR, estimated glomerular filtration rate.

**Figure 4:**
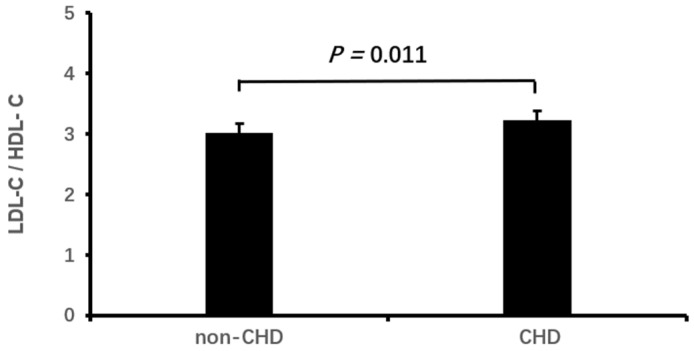
The LDL-C/HDL-C ratio in patients with and without CHD. LDL-C, low-density lipoprotein cholesterol; HDL-C, high-density lipoprotein cholesterol; CHD, coronary heart disease.

**Figure 5:**
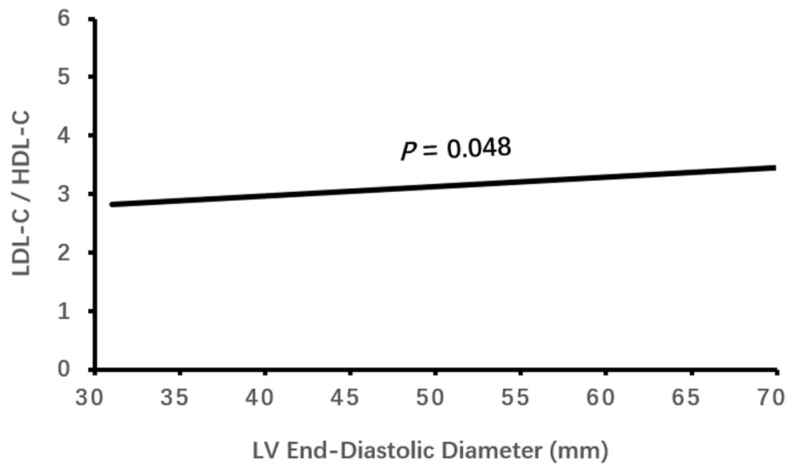
The association of LDL-C/HDL-C ratio with LV end-diastolic diameter. LDL-C, low-density lipoprotein cholesterol; HDL-C, high-density lipoprotein cholesterol; LV, left ventricular.

Logistic binary regression analysis of the high LDL-C/HDL-C ratio (≥3.33) with clinical characteristics and echocardiographic parameters showed that a high LDL-C/HDL-C ratio was associated independently with male gender, elevated HbA1c, decreased eGFR, CHD and enlarged LVEDD (all *p* < 0.05, Table 3, Figure 1, Figure 2, Figure 3, Figure 4 and Figure 5).

## 4. Discussion

In aged patients with hypercholesterolemia, a high LDL-C/HDL-C ratio was correlated with male gender, high HbA1c, low eGFR, CHD and left ventricular enlargement.

HDL is responsible for the transportation of cholesterol from peripheral tissues to the liver for degradation of the cholesterol. It has beneficial effects such as anti-atherosclerosis, anti-inflammation, anti-oxidative stress and inhibition of thrombosis, whereas LDL has the opposite roles [8,22]. Although blood HDL-C is decreased markedly in post-menopausal women [23], it is still much higher than that in men of the same age. Furthermore, blood LDL-C is remarkably higher in post-menopausal women than in women of childbearing age. However, LDL-C levels were lower in women as compared with men at the same age [20,24]. The quick decrease in estrogen in post-menopausal women leads to the activation of a cholesterol synthesis rate-limiting enzyme (3-hydroxy-3-methyl glutaryl coenzyme A reductase). Then, the production of cholesterol is increased among post-menopausal women. Meanwhile, the hepatic LDL receptor expression declines, which reduces the clearance of plasma LDL-C, ultimately leading to a significant elevation in blood LDL-C in post-menopausal women [25,26]. However, it is not well known if HDL-C is increased or decreased in the aging female population [23,27]. Nevertheless, studies have demonstrated that hormone replacement therapy with estrogen and progesterone reduced LDL-C while elevating HDL-C [28].

Although dietary intake of exogenous cholesterol is less than one-third of the cholesterol synthesized in the human body, studies have confirmed that dietary sources of cholesterol are significantly associated with elevated blood LDL-C. In addition, a study showed that dietary cholesterol is negatively correlated with HDL-C in men but positively associated with HDL-C in women [29]. Furthermore, it was observed that LDL-C in men had little change with increasing age; Nevertheless, the LDL-C in men was consistently higher than that in women at the corresponding age [30]. Moreover, males often have unhealthy lifestyles including high-fat diets, cigarette smoking, and alcohol abuse, which will exacerbate the decrease in HDL-C [31,32]. Consequently, men would often have a higher LDL-C/HDL-C ratio than women. In this study, we found that a high LDL-C/HDL-C ratio was more common in elderly men than women among hypercholesterolemic patients. This finding is consistent with the previous report [30].

In the case of dyslipidemia, the accumulated fat in the tissue extracellular mesenchyme reduces cellular sensitivity to insulin and triggers insulin resistance, leading to elevated blood glucose and HbA1c and the onset of type 2 DM [8]. Studies have confirmed the positive correlation between LDL-C and hyperglycemia [33], whereas HDL-C and apoA1 were associated reversely with hyperglycemia and increased HbA1c [34,35]. It was found that apoB100/apoA1 was positively associated with HbA1c [36], and the respective association of the LDL-C/HDL-C ratio with incident type 2 DM has been confirmed [8]. However, to the best of our knowledge, we are the first to find that a high LDL-C/HDL-C ratio was associated with increased HbA1c in the elderly with hypercholesterolemia.

Small dense LDL-C has a low affinity for its receptor, which makes it slower to be removed from blood circulation. It is susceptible to being oxidized and leading to cellular damage [37]. Excessive blood LDL-C, especially oxidized LDL-C, is able to be retained in glomerular podocytes and destroy the glomerular filtration membrane, resulting in glomerular epithelial apoptosis, necrosis and renal dysfunction. Studies have proved that high LDL-C and low HDL-C were associated with a decline in eGFR [38,39,40,41]. Additionally, a high LDL-C/HDL-C ratio has been associated with decreased eGFR and renal function [9]. The decline in eGFR could result from high-LDL-C- and low-HDL-C-mediated renal artery atherosclerotic stenosis and ischemic kidney injury [42,43]. In the present study, we found that a high LDL-C/HDL-C ratio was significantly associated with decreased eGFR in elderly with hypercholesterolemia. This result is consistent with the previous study [9].

Small dense oxidized LDL in the blood can also inhibit HDL’s functions and lead to vascular endothelial dysfunction and cardiomyocytic apoptosis and necrosis. LDL-C is susceptible to accumulation under the endothelium of the coronary artery wall. Mononuclear macrophages invade coronary artery walls, leading to coronary atherosclerosis and thrombosis [44], which lead to myocardial ischemia, infarction and cardiac events [45,46]. Studies have confirmed that a high LDL-C/HDL-C ratio was correlated with prevalent CHD and cardiac remodeling [10,45]. Meanwhile, previous studies have confirmed a significant correlation between low eGFR and left ventricular remodeling [47,48]. In this study, we demonstrated that a high LDL-C/HDL-C ratio is associated with low eGFR, CHD and left ventricular enlargement in elderly with hypercholesterolemia. Our finding is consistent with the previous reports [10,45,47]. This study included more women than men, as older women are prone to hypercholesterolemia [49].

## 5. Limitations

The main limitation of this study is that it was conducted in a single tertiary medical center, instead of a community. Moreover, this study was based on retrospectively collected cross-sectional data from a Chinese elderly population. This kind of cross-sectional study setting is prone to selection bias. Thus, although the observed relationship between LDL-C/HDL-C ratio and metabolic and cardiac parameters showed a correlation, similar studies on different races including elderly populations are needed to reconfirm the causal relationship between these parameters. Therefore, the observed associations of the LDL-C/HDL-C ratio with male gender, high HbA1c, low eGFR, CHD and cardiac remodeling need to be proved by further investigations in other elderly populations.

## 6. Conclusions

In elderly with hypercholesterolemia, our study suggested that a high LDL-C/HDL-C ratio is associated with left ventricular remodeling, possibly through CHD and low eGFR. A high LDL-C/HDL-C ratio can be used to predict subclinical adverse cardiac remodeling.

## Figures and Tables

**Table 1:** Comparison of clinical characteristics among tertiles of LDL-C/HDL-C ratio (LHR).

	Low LHR	Moderate LHR	High LHR	*r*	*p*
	LHR < 2.63	2.63 ≤ LHR < 3.33	LHR ≥ 3.33		
	*n* = 376	*n* = 376	*n* = 377		
LHR	2.22 ± 0.29	2.98 ± 0.20 *	3.99 ± 0.72 *^†^	1.000	<0.001
Male, *n* (%)	85 (22.6)	100 (26.6)	141 (37.4) *^†^	0.133	<0.001
Age, year	75.50 ± 6.51	75.14 ± 7.26	74.69 ± 6.68	−0.049	0.240
Body mass index, kg/m^2^	23.20 ± 3.63	24.43 ± 3.38 *	24.69 ± 3.17 *	0.176	<0.001
Smoking, *n* (%)	44 (11.9)	49 (13.2)	90 (24.2) *^†^	0.136	<0.001
Drinking, *n* (%)	43 (11.6)	47 (12.7)	68 (18.3) *	0.078	0.019
Type 2 DM, *n* (%)	95 (25.3)	128 (34.0) *	159 (42.2) *	0.146	<0.001
Diabetic duration, year	1.74 ± 4.62	2.88 ± 6.39 *	3.67 ± 6.81 *	0.131	<0.001
Hypertension, *n* (%)	243 (64.6)	250 (66.5)	277 (73.5) *	0.078	0.023
Hypertensive duration, year	7.62 ± 10.15	8.18 ± 11.24	8.28 ± 10.53	0.025	0.654
Systolic BP, mmHg	142.43 ± 22.52	143.11 ± 21.37	144.64 ± 21.58	0.041	0.365
Diastolic BP, mmHg	76.48 ± 12.65	77.51 ± 12.11	79.23 ± 13.40 *	0.088	0.011
Fatty liver, *n* (%)	66 (22.8)	105 (38.9) *	107 (39.6) *	0.147	<0.001
Coronary heart disease, *n* (%)	67 (17.8)	78 (20.7)	108 (28.6) *^†^	0.106	0.001
Stroke, *n* (%)	141 (37.5)	129 (34.3)	126 (33.4)	−0.035	0.468
HbA1c, %	6.12 ± 1.04	6.51 ± 1.45 *	6.81 ± 1.76 *^†^	0.190	<0.001
eGFR, mL/min·1.73 m^2^	73.63 ± 18.40	73.71 ± 19.53	68.65 ± 21.85 *^†^	−0.101	0.001
hsCRP, mg/L	2.01 ± 2.79	2.29 ± 2.94	2.81 ± 3.27 *	0.109	0.002
LDL-C, mmol/L	3.77 ± 0.35	3.91 ± 0.46 *	4.19 ± 0.68 *^†^	0.314	<0.001
HDL-C, mmol/L	1.73 ± 0.28	1.32 ± 0.16 *	1.07 ± 0.18 *^†^	−0.776	<0.001
TC, mmol/L	5.75 ± 0.56	5.62 ± 0.62 *	5.87 ± 0.89 *^†^	0.067	<0.001
TG, mmol/L	1.33 ± 0.60	1.70 ± 0.73 *	2.20 ± 1.18 *^†^	0.377	<0.001

Values presented as mean ± SD or *n* (%). DM, diabetes mellitus; BP, blood pressure; HbA1c, glycated hemoglobin; eGFR, estimated glomerular filtration rate; hsCRP, high-sensitivity C-reactive protein; LDL-C, low-density lipoprotein cholesterol; HDL-C, high-density lipoprotein cholesterol; TC, total cholesterol; TG, triglyceride; * *p* < 0.05 versus low LHR group; ^†^ *p* < 0.05 versus moderate LHR group.

**Table 2:** Comparison of echocardiographic parameters among tertiles of LDL-C/HDL-C ratio (LHR).

	Low LHR	Moderate LHR	High LHR	*r*	*p*
	LHR < 2.63	2.63 ≤ LHR < 3.33	LHR ≥ 3.33		
	*n* = 376	*n* = 376	*n* = 377		
RAD, mm	33.32 ± 3.63	33.39 ± 3.54	33.72 ± 3.98	0.045	0.276
RVD, mm	18.85 ± 1.65	19.13 ± 1.57 *	19.26 ± 1.75 *	0.102	0.002
LAD, mm	29.51 ± 4.69	30.42 ± 4.62 *	31.22 ± 5.55 *^†^	0.139	<0.001
LVESD, mm	30.48 ± 5.16	30.60 ± 4.31	31.71 ± 6.01 *^†^	0.095	0.005
LVEDD, mm	45.92 ± 5.04	46.09 ± 4.69	46.87 ± 5.77 *^†^	0.075	0.027
IVST, mm	10.52 ± 1.20	10.66 ± 1.22	10.90 ± 1.32 *^†^	0.123	<0.001
LVPWT, mm	10.36 ± 1.08	10.49 ± 1.18	10.68 ± 1.25 *^†^	0.110	0.001
LVEF, %	62.61 ± 6.40	62.66 ± 5.28	61.06 ± 7.35 *^†^	−0.099	0.001
E/A < 1, *n* (%)	336 (100)	347 (99.4)	334 (98.5)	−0.073	0.064

Values presented as mean ± SD. LDL-C, low-density lipoprotein cholesterol; HDL-C, high-density lipoprotein cholesterol ratio; RAD, right atrial diameter; RVD, right ventricular diameter; LAD, left atrial diameter; LVESD, left ventricular end-systolic diameter; LVEDD, left ventricular end-diastolic diameter; IVST, interventricular septal thickness; LVPWT, left ventricular posterior wall thickness; LVEF, left ventricular ejection fraction; E/A, peak early (E)/late (A) filling velocities; * *p* < 0.05 versus low LHR group; ^†^ *p* < 0.05 versus moderate LHR group.

**Table 3:** Regression analysis of parameters associated with LDL-C/HDL-C ratio ≥ 3.33.

	β	SE	Wald χ^2^	*p*	OR (95% CI)
Male	0.690	0.193	12.848	<0.001	1.994 (1.367–2.909)
Type 2 DM	−0.024	0.217	0.012	0.911	0.976 (0.638–1.493)
Hypertension	−0.012	0.204	0.003	0.955	0.989 (0.662–1.476)
Body mass index	0.048	0.028	2.971	0.085	1.049 (0.993–1.017)
Diastolic BP	0.002	0.007	0.234	0.628	1.003 (0.990–1.017)
Fatty liver	0.368	0.192	3.679	0.055	1.445 (0.992–2.106)
Coronary heart disease	0.547	0.215	6.471	0.011	1.727 (1.135–2.632)
HbA1c	0.233	0.071	10.660	0.001	1.262 (1.097–1.451)
eGFR	−0.009	0.004	4.243	0.039	0.991 (0.983–1.000)
hsCRP	0.016	0.025	0.381	0.537	1.016 (0.967–1.068)
RVD	0.026	0.061	0.189	0.664	1.027 (0.911–1.157)
LAD	−0.005	0.024	0.041	0.840	0.995 (0.950–1.043)
LVESD	0.158	0.102	2.391	0.122	1.171 (0.757–1.430)
LVEDD	−0.139	0.071	3.819	0.048	0.870 (0.756–1.000)
IVST	0.074	0.115	0.412	0.521	1.077 (0.859–1.349)
LVPWT	0.043	0.123	0.120	0.729	1.044 (0.820–1.329)
LVEF	0.024	0.040	0.351	0.553	1.024 (0.947–1.108)

LDL-C, low-density lipoprotein cholesterol; HDL-C, high-density lipoprotein cholesterol ratio; β, regression coefficient; SE, standard error; Wald, Chi-square value; OR, odds ratio; CI, confidence interval; DM, diabetes mellitus; BP, blood pressure; HbA1c, glycated hemoglobin; eGFR, estimated glomerular filtration rate; hsCRP, high-sensitivity C-reactive protein.; RVD, right ventricular diameter; LAD, left atrial diameter; LVESD, left ventricular end-systolic diameter; LVEDD, left ventricular end-diastolic diameter; IVST, interventricular septal thickness; LVPWT, left ventricular posterior wall thickness; LVEF, left ventricular ejection fraction.

## Data Availability

All data generated or analyzed during this study are included in this article, along with references to data from cited published studies. The database is not publicly available. Further inquiries can be directed to the corresponding author.
